# Association between Time to Emergent Surgery and Outcomes in Trauma Patients: A 10-Year Multicenter Study

**DOI:** 10.3390/medicina60060960

**Published:** 2024-06-10

**Authors:** Chi-Hsuan Tsai, Meng-Yu Wu, Da-Sen Chien, Po-Chen Lin, Jui-Yuan Chung, Chi-Yuan Liu, I-Shiang Tzeng, Yueh-Tseng Hou, Yu-Long Chen, Giou-Teng Yiang

**Affiliations:** 1 Department of Emergency Medicine, Taipei Tzu Chi Hospital, Buddhist Tzu Chi Medical Foundation, New Taipei 231, Taiwan; 2 Department of Emergency Medicine, School of Medicine, Tzu Chi University, Hualien 970, Taiwan; 3 Graduate Institute of Injury Prevention and Control, Taipei Medical University, Taipei 231, Taiwan; 4 Department of Emergency Medicine, Cathay General Hospital, Taipei 106, Taiwan; 5 School of Medicine, Fu Jen Catholic University, Taipei 242, Taiwan; 6 School of Medicine, National Tsing Hua University, Hsinchu 300, Taiwan; 7 Department of Orthopedic Surgery, Taipei Tzu Chi Hospital, Buddhist Tzu Chi Medical Foundation, New Taipei 231, Taiwan; 8 Department of Orthopedics, School of Medicine, Tzu Chi University, Hualien 970, Taiwan; 9 Department of Research, Taipei Tzu Chi Hospital, Buddhist Tzu Chi Medical Foundation, New Taipei 970, Taiwan

**Keywords:** golden hour, time to definitive care, mortality, trauma

## Abstract

*Background*: Research on the impact of reduced time to emergent surgery in trauma patients has yielded inconsistent results. Therefore, this study investigated the relationship between waiting emergent surgery time (WEST) and outcomes in trauma patients. *Methods*: This retrospective, multicenter study used data from the Tzu Chi Hospital trauma database. The primary clinical outcomes were in-hospital mortality, intensive care unit (ICU) admission, and prolonged hospital length of stay (LOS) of ≥30 days. *Results*: A total of 15,164 patients were analyzed. The median WEST was 444 min, with an interquartile range (IQR) of 248–848 min for all patients. Patients who died in the hospital had a shorter median WEST than did those who survived (240 vs. 446 min, *p* < 0.001). Among the trauma patients with a WEST of <2 h, the median time was 79 min (IQR = 50–100 min). No significant difference in WEST was observed between the survival and mortality groups for patients with a WEST of <120 min (median WEST: 85 vs. 78 min, *p* < 0.001). Multivariable logistic regression analysis revealed that WEST was not associated with an increased risk of in-hospital mortality (adjusted odds ratio [aOR] = 1.05, 95% confidence interval [CI] = 0.17–6.35 for 30 min ≤ WEST < 60 min; aOR = 1.12, 95% CI = 0.22–5.70 for 60 min ≤ WEST < 90 min; and aOR = 0.60, 95% CI = 0.13–2.74 for WEST ≥ 90 min). *Conclusions*: Our findings do not support the “golden hour” concept because no association was identified between the time to definitive care and in-hospital mortality, ICU admission, and prolonged hospital stay of ≥30 days.

## 1. Introduction

Traumatic injuries present a substantial global threat; they contribute considerably to global morbidity and mortality. Early definite care is crucial for high-risk trauma patients, with interventions for such patients including surgery [1,2,3,4,5,6]. According to the “golden hour” concept, the first hour following a traumatic injury is the most crucial [7].

The term “golden hour” is often attributed to R. Adams Cowley, the founder of Baltimore’s Shock Trauma Institute. In a 1975 article, Cowley asserted that “the first hour after injury will largely determine a critically injured person’s chances for survival”. The golden hour concept emphasizes that critically injured patients must receive definitive care within 60 min of sustaining injuries. During this crucial period, immediate medical attention can profoundly influence a patient’s prognosis. Swift and effective intervention within this timeframe often determines whether a patient will live or die or will experience full recovery or lasting disability. Prompt assessment, stabilization, and transport to appropriate medical facilities during this hour are essential to reduce complications and improve survival and recovery outcomes. Providing medical care within the golden hour is imperative because delays can lead to worsened outcomes and increased mortality rates. The golden hour concept was proposed in the 1970s; it has not been supported by empirical data or research but has gained widespread acceptance because of its clinical plausibility. Therefore, its validity remains unclear.

Trauma patients requiring emergent surgery may benefit from prompt surgical intervention, as suggested by the golden hour concept, with such intervention potentially improving their prognosis. However, research on the relationship between the waiting emergent surgery time (WEST) and trauma patient outcomes has produced inconsistent findings. Most studies investigating the impact of reduced surgical wait times have focused on patients with hip fracture, and they have obtained varying results. Additionally, research efforts have often been limited by small sample sizes or a primary emphasis on reducing time intervals rather than improving patient outcomes.

Gaining a comprehensive understanding of the golden hour concept is imperative. In Taiwan, level 1 trauma centers, which are accredited on the basis of their emergency capacity, have attending surgeons available 24/7 for all major trauma resuscitations. These centers also provide resuscitation interventions such as transcatheter arterial embolization or surgery, even on weekends and holidays [6,8]. Although studies have reported the optimal time to definitive care to be 2 h for procedures such as exploratory laparotomy and craniotomy at level 1 hospitals, in Taiwan, the time limit for emergent trauma surgeries is typically 30 min [9,10]. The current study investigated the relationship between the time to definitive care and trauma patient outcomes.

## 2. Methods

### 2.1. Study Design and Setting

This study was a retrospective cohort analysis of data from the Tzu Chi Hospital trauma registry, and the study protocol was approved by the Institutional Review Board of Taipei Tzu Chi Hospital (IRB number: 12-XD-077). The trauma database of Tzu Chi Hospital is a collaborative effort among four hospitals within the Tzu Chi Hospital system, with the hospitals located in Hualien, Taipei, Taichung, and Dalin. This database includes the data of patients admitted with trauma-related conditions identified by International Classification of Diseases, Ninth Revision, Clinical Modification codes 800–959 (excluding 905–909 and 930–939) or International Classification of Diseases, Tenth Revision, Clinical Modification codes S00–T98 (excluding T15–T19 and T90–T98) as well as those with major traumatic injuries. The database includes information regarding a comprehensive set of 152 variables associated with trauma, covering aspects such as demographics, injury mechanisms, injury types, injury severity, vital signs, surgical interventions, and in-hospital mortality. The current study adhered to the Strengthening the Reporting of Observational Studies in Epidemiology (STROBE) guidelines (Supplementary Table S1) [11].

### 2.2. Participant Selection

**Figure 1:**
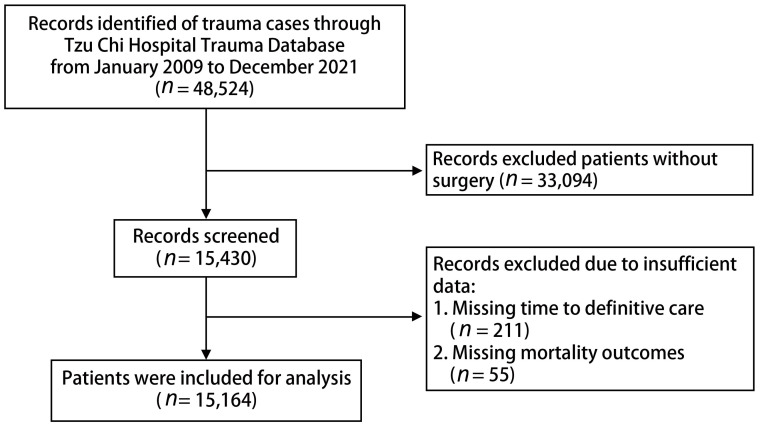
Flow diagram of participant selection.

This study included patients listed on the Tzu Chi Hospital trauma database between January 2009 and December 2021. Initially, 48,524 patients were considered for analysis. However, patients who did not undergo surgery (n = 33,094), those without a recorded time to definitive care (n = 211), and those without data on mortality outcomes (n = 55) were excluded. Ultimately, 15,164 patients were included in the analysis. A detailed flow diagram of the participant selection process is presented in Figure 1.

### 2.3. Variable Measurements

The study analyzed the fundamental characteristics of the trauma cohort, including age, sex, pre-existing medical conditions, emergency triage classification, vital signs, injury etiology, and injury severity. Vital parameters, such as heart rate (HR), systolic blood pressure (SBP), diastolic blood pressure (DBP), and respiratory rate (RR), were documented. Trauma severity was assessed using the injury severity score (ISS) and the revised trauma score (RTS). The ISS is used to evaluate the severity of multiple injuries, with scores from various body regions (head, chest, abdomen, extremities, and other areas) combined into a single value ranging from 0 to 75, with higher scores indicating more severe trauma [12]. The RTS is used to assess trauma severity on the basis of a patient’s physiological parameters, including SBP, RR, and level of consciousness [13]. These scoring systems are critical for the initial assessment and classification of trauma patients; they assist health-care providers in quickly assessing injury severity and developing appropriate treatment plans. Use of the ISS and RTS can help improve survival rates and patient outcomes as well as assist heal-care providers in effectively allocating emergency and medical resources. In the current study, patients with an ISS ≥ 16 or an RTS < 7 were defined as the major trauma population. The patients were categorized into two groups: those with traumatic brain injury (TBI, head Abbreviated Injury Score [AIS] ≥ 3) and those without TBI (head AIS score < 3). For the subgroup analysis, the geriatric population was defined as individuals aged ≥ 65 years. The mechanisms of injury included motor vehicle collision, low falls (falling from < 1 m), high falls (falling from ≥ 1 m), and others (such as drowning, burns, and cold injuries).

The primary variable in this study was each patient’s time to definitive care, defined as the time to surgical intervention for acute trauma injury. Time to emergent surgery was calculated as the interval from the patient’s arrival at the hospital to the start of surgical intervention. We categorized the WEST into 30 min intervals. Although emergency intervention is generally defined as that occurring within 1 h of injury, studies have indicated that the optimal time to definitive care for most emergent trauma interventions may extend up to 2 h, with this timeframe employed in some level 1 trauma centers [9,14]. To account for this, we conducted a sensitivity analysis focusing on patients who received emergent surgery within 2 h of injury.

### 2.4. Clinical Outcomes

We focused on three primary clinical outcomes: in-hospital mortality, admission to the intensive care unit (ICU), and extended hospitalization, defined as a length of stay (LOS) of ≥30 days. Additionally, we evaluated the frequency of ICU readmissions, the duration of ICU stays, extended ICU LOS (specified as an ICU stay exceeding 14 days), and the total duration of hospitalization for each patient.

### 2.5. Statistical Analysis

We conducted a comprehensive statistical analysis of all demographic information, injury details, and clinical outcomes by using SPSS software (version 20.0, SPSS, Chicago, IL, USA). The distribution patterns of continuous data were assessed using the Kolmogorov–Smirnov test. Continuous variables are presented as medians with interquartile ranges (IQRs), and categorical variables are presented as counts and percentages. Continuous data were analyzed using either nonparametric analysis of variance or the Mann–Whitney U test. Categorical and nominal data were evaluated using Pearson’s chi-square test or Fisher’s exact test. The relationship between the scoring systems and the three major trauma patient outcomes was determined using multivariable logistic regression. The factors considered in the regression analysis were variables with a *p* value < 0.10 in the chi-square or Mann–Whitney U tests or those of clinical significance, such as age, sex, injury type, and injury mechanism, by using a forced entry method. Sensitivity analyses were conducted to evaluate the associations across different groups, including categories of injury severity (minor injury: ISS < 16 or RTS ≥ 7 and major injury: ISS ≥ 16 or RTS < 7), age groups (nongeriatric: age < 65 years and geriatric: age ≥ 65 years), WEST durations (WEST < 30 min, 30 min ≤ WEST < 60 min, 60 min ≤ WEST < 90 min, and WEST ≥ 90 min), and major injury sites (head AIS ≥ 3, chest AIS ≥ 3, and abdominal AIS ≥ 3). All tests were two-sided, with significance set at *p* < 0.05.

## 3. Results

### 3.1. Characteristics of Study Participants

The in-hospital mortality rate, ICU admission rate, and rate of prolonged hospital LOS of ≥30 days were 1.0%, 12.0%, and 4.5%, respectively. Table 1 presents the demographic characteristics of all patients. The geriatric population (age ≥ 65 years) accounted for 41.4% and 63.4% of the mortality group. Patients in the in-hospital mortality group had higher triage levels, with 49.7% classified as triage level I. Penetrative injuries were present in only 5.3% of all patients. Cardiovascular disease was the most common chronic condition, accounting for 27.6% of the conditions, followed by diabetes (15.9%). Major injuries (ISS ≥ 16) were present in 7.4% of all patients and 67.6% of the in-hospital mortality group. Low falls were the most common injury mechanism, accounting for 39.9% of the injuries, followed by road traffic injuries, at 37.7%. TBI occurred in 6.7% of all patients and in 58.6% of the in-hospital mortality group.

### 3.2. Time to Emergent Surgery in Subgroups

Table 2 presents the results of comparisons of the WEST among all patients across various subgroups. The median WEST for all patients was 444 min, with an IQR of 248–848 min. Patients who died in hospital had a notably shorter WEST than survivors did (median WEST: 240 vs. 446 min, *p* < 0.001). After stratification by other patient characteristics, a significantly shorter WEST was observed among patients younger than 65 years (median WEST: 419 vs. 478 min, *p* < 0.001), those with major injuries to the abdomen (median WEST in patients with abdominal AIS ≥ 3 vs. head AIS ≥ 3 vs. chest AIS ≥ 3: 353 vs. 413 vs. 492 min, *p* < 0.001), those with major injuries (ISS ≥ 16 or RTS < 7; median WEST in patients with RTS < 7 vs. RTS ≥ 7 and ISS ≥ 16 vs. ISS < 16: 301 vs. 450 min and 397 vs. 447 min, respectively, *p* < 0.001), and those with a hospital stay exceeding 30 days (median WEST: 446 vs. 406 min, *p* < 0.001).

### 3.3. Trauma Patients with WEST Less than 2 h

According to previous studies, many level 1 trauma centers have implemented the optimal time interval of 2 h for emergent trauma interventions [9,14]. Table 3 presents the results of sensitivity analyses for trauma patients who received emergent surgery within 2 h. Among the trauma patients with a WEST of <2 h, the median time was 79 min, with an IQR of 50–100 min. No significant difference in WEST was noted between the survival and mortality groups of patients with a WEST of <120 min (median WEST: 85 vs. 78 min, *p* < 0.001). The patients aged ≥ 65 years (median WEST: 81 vs. 72 min, *p* < 0.001), those with minor injuries (ISS < 16 or RTS ≥ 7; median WEST in patients with RTS ≥ 7 vs. RTS < 7 and ISS < 16 vs. ISS ≥ 16: 76 vs. 56 min and 88 vs. 76 min, respectively, *p* < 0.001), those with major injuries to the chest (median WEST in patients with chest AIS ≥ 3 vs. head AIS ≥ 3 vs. abdominal AIS ≥ 3: 77 vs. 91 vs. 81 min, *p* < 0.001), and those without ICU admission (median WEST: 76 vs. 87 min, *p* < 0.001) had a shorter WEST.

### 3.4. Association between WEST and Clinical Outcomes

**Figure 2:**
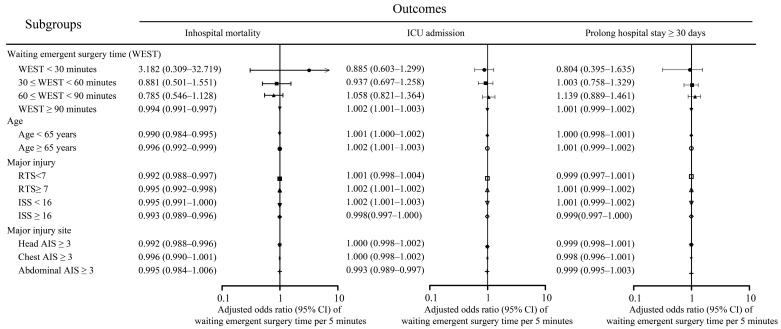
Subgroup analysis illustrating association between a 5 min increase in waiting emergent surgery time (WEST) and in-hospital mortality, ICU admission, and prolonged hospital stay (≥30 days) across various subgroups.

The results of the multivariable logistic regression in Table 4, which included all patient data, revealed several variables to be significantly associated with increased odds of mortality. These variables included age ≥ 65 years (adjusted odds ratio [aOR] = 3.62), male sex (aOR = 2.12), RTS < 7 (aOR = 5.15), and ISS ≥ 16 (aOR = 8.29). Notably, WEST was not correlated with an elevated risk of in-hospital mortality. Additionally, no significant association was noted between WEST and ICU admission or prolonged hospital stay of ≥30 days. The results of the subgroup analysis (Figure 2) indicated that a longer WEST was associated with a reduced risk of in-hospital mortality in patients with WEST ≥ 90 min (aOR = 0.994, 95% confidence interval [CI] = 0.991–0.997) in those aged < 65 years (aOR = 0.990, 95% CI = 0.984–0.995) and ≥65 years (aOR = 0.996, 95% CI = 0.992–0.999), as well as in those with minor injuries (RTS ≥ 7: aOR = 0.995, 95% CI = 0.992–0.998; ISS < 16: aOR = 0.995, 95% CI = 0.991–1.000) or major injuries (RTS < 7: aOR = 0.992, 95% CI = 0.988–0.997; ISS ≥ 16: aOR = 0.993, 95% CI = 0.989–0.996), and those with head AIS ≥ 3 (aOR = 0.992, 95% CI = 0.988–0.996).

## 4. Discussion

Our findings revealed no association between WEST and in-hospital mortality, ICU admission, and prolonged hospital stay (≥30 days). However, the subgroup analysis indicated that a longer WEST (per 5 min) was associated with increased survival in patients with a WEST ≥ 90 min, those aged < 65 years, those aged ≥ 65 years, those with minor injuries (RTS ≥ 7 and ISS < 16), those with major injuries (RTS < 7 or ISS ≥ 16), and those with major injuries to the head (head AIS ≥ 3). Additionally, a longer WEST was associated with an increased risk of ICU admission in patients with a WEST ≥ 90 min, those aged ≥ 65 years, those with minor injuries, and those with major injuries in the abdomen.

Previous studies investigating the relationship between waiting time for surgery and mortality and functional outcomes have predominantly focused on fracture surgeries. For example, the TRON Study, a propensity score–matched multicenter investigation involving 779 patients who underwent ankle fracture surgery, revealed significantly longer operative times and higher infection rates in their delayed operation group compared with their early operation group [15]. Pincus et al. [16] analyzed 42,230 patients with hip fractures and revealed that prolonged wait times were associated with an increased risk of 30-day mortality and other complications. The time window for waiting for surgery is considerably narrower for complex fractures and major traumatic injuries than it is for simple fractures. Several studies have provided support for the golden hour concept with respect to emergent surgery. Hsieh et al. analyzed the data of 963 trauma patients from the Pan-Asian Trauma Outcome Study registry and revealed a positive association between a shorter time to definitive care within 2 h and patient survival and functional outcomes [14]. Another observational study that used data from the Trauma and Audit Research Network revealed that trauma patients who underwent secondary transfer experienced a prolonged time to urgent surgery and increased crude mortality rates [17]. However, our study did not obtain evidence supporting these associations, even among the subgroups with major trauma, TBI, major chest injuries, and torso injuries. The relationship between WEST and trauma outcomes might be influenced by presurgical treatments, which can attenuate the effect of WEST and render the effect nonsignificant. This underscores the importance of actively promoting recovery during the waiting period for surgery through interventions such as proactive blood and fluid transfusions, administration of hemostatic medications, maintenance of body temperature, and ensuring adequate ventilation to prevent acidosis [18,19]. Furthermore, the number and type of presurgical treatments can influence the time to emergent surgery. Surgeons and anesthesiologists generally consider stable baseline blood pressure and HR to be necessary for effective surgery. Conservative trauma practitioners may even advocate for ensuring acceptable blood pressure levels before surgery to avoid potential complications associated with operating on hemodynamically unstable patients. Therefore, patients with major trauma might experience a slightly higher WEST than those with minor injuries do; this extended WEST may be partially attributable to implementation of preoperative stabilization treatments and may reflect the greater complexity of injuries requiring more interventions. Nevertheless, studies investigating WEST and patient outcomes have failed to account for the potential confounding effect of presurgical treatments, which may have introduced bias into their results [14,17].

In our study, a subgroup analysis focusing on patients with major head, chest, and abdominal injuries revealed that longer intervals prior to surgical intervention did not increase the risk of mortality in the TBI population, nor did it have any association with the outcomes of patients with major chest or abdominal injuries. This finding contrasts with those of previous research primarily focused on TBIs [20,21]. A key reason for this inconsistency is that the location and severity of head trauma exert a more substantial influence on clinical outcomes than the timing of surgical procedures does. In clinical practice, neurosurgeons typically evaluate the prognoses of patients with head trauma and recommend surgery accordingly. For patients with severe injuries, surgical intervention may not significantly influence outcomes, whereas for those with less severe injuries, immediate surgery may not be required. Instead, the patient may be observed to determine whether surgical intervention is necessary as the condition evolves. Therefore, the inclusion of observation time in calculations of WEST for patients with TBI may introduce bias. Considering patients who are under observation before surgery in combination with those who require immediate surgery, such as those with epidural hematomas, may lead to findings indicating lower in-hospital mortality rates and longer intervals to definitive care. This underscores the influence of patient heterogeneity on outcomes and indicates that certain types of TBI may not require immediate intervention. Consistent with our findings, a meta-analysis of 16 studies revealed that patient outcomes were not significantly influenced by the timing of surgery in 68.7% of the included studies. Moreover, the effect of time to surgery on outcomes was not significant in the majority (75%) of studies focusing on patients with severe TBI [22]. In the current study, we considered different TBI severities (mild, moderate, and severe). The results indicated that shorter intervals to emergent surgery did not reduce in-hospital mortality in mild or severe TBI populations (mild TBI: aOR = 0.993, 95% CI = 0.987–0.999; moderate TBI: aOR = 0.997, 95% CI = 0.988–1.006; severe TBI: aOR = 0.992, 95% CI = 0.985–0.999). Future studies should conduct in-depth analyses of various traumatic intracranial hemorrhage types and include parameters such as the bleeding volume and brain herniation to minimize bias.

This study has several strengths. First, it validated the golden hour concept for surgical intervention in trauma patients. This finding holds clinical importance because it underscores the importance of timely stabilization of injury conditions and adequate resuscitation. Second, we conducted many subgroup analyses to investigate the association between WEST and clinical outcomes. These analyses confirmed that a shorter WEST did not significantly reduce the risk of mortality in trauma patients, even in those with major trauma injuries. This indicates that adequate resuscitation may be more crucial than shortening the WEST is.

This study has some limitations that should be considered. First, because this was a retrospective study, data may be missing, which may have introduced bias. The unmeasured variables in this study may also have influenced the study results. Moreover, our exclusion of unreasonable data points and outliers may have introduced selection bias into the final dataset. Although a randomized controlled trial could address these limitations, ethical considerations might render such a trial infeasible. The majority of the excluded patients lacked documentation for critical variables such as time to definitive care and mortality outcomes, which are crucial aspects of our analysis. Furthermore, imputing missing data for time to definitive care and mortality outcome data would be inappropriate because of the considerable variability in these measures related to factors such as hospital capacity, injury severity, and personalized treatment plans. Imputation under these circumstances could lead to inaccurate estimations. Additionally, imputing missing data for other variables would not notably improve the accuracy of the analysis. To mitigate these limitations, we compared the baseline characteristics between the included patients and patients excluded because of missing data (refer to Supplementary Table S2). Significant differences were noted between the two groups, particularly for outcome variables such as in-hospital mortality, ICU admission, and prolonged hospitalization. However, the in-hospital mortality rates were not statistically different between the included and excluded samples. Moreover, some missing data for time to definitive care may be considered “missing at random” because case managers may not have accurately predicted patient outcomes when documenting this variable. Second, emergency medical system dispatch times can considerably influence a patient’s WEST and clinical outcomes. However, because prehospital transport times in Taiwan are generally < 20 min, we excluded this variable from the analysis; in Taipei, the median transport interval is 7 min, and the median prehospital interval is 23 min, which are significantly shorter than those in other countries [23]. Third, our study excluded patients who did not receive surgical intervention. However, we were unable to determine the reasons for these patients ultimately not undergoing surgery. Possible reasons include ‘Do not resuscitate’ orders and poor prognosis leading to palliative care. Fourth, although the severity of injury is a major determinant of mortality among trauma patients, surgeons employing a proactive approach may also influence patient outcomes. Currently, no objective assessment method is available for this surgeon-specific factor. Fifth, missing data on resuscitation interventions for trauma patients, such as fluid resuscitation volume, blood transfusion, transcatheter arterial embolization, and emergent thoracotomy, may have influenced the study outcomes. These interventions can prolong the WEST, and accounting for them is crucial to accurately evaluating the effect of time to surgery. However, current studies supporting the golden hour concept often lack adjustment for these variables [14,24]. Finally, our findings may not be applicable to the general population. Major injuries were uncommon in our data (7.4%), with blunt injuries being the most prevalent (5.3%). Falls were the leading cause of injury (50%), with high falls accounting for 10.1% and lower falls accounting for 39.9% of the injuries. Further research involving broader populations and robustly controlling for confounding factors is required to validate our findings.

## 5. Conclusions

Our findings revealed no significant association between time to emergent surgery and in-hospital mortality, ICU admission, or prolonged hospital stays of ≥30 days, which contradicts the golden hour concept. Furthermore, our subgroup analysis revealed that a longer WEST (per 5 min) was associated with increased survival for patients with a WEST of ≥90 min, regardless of age group (<65 and ≥65 years), injury severity (minor injury with RTS ≥ 7 and ISS < 16 and major injury with RTS < 7 or ISS ≥ 16), and injury site (major injuries to the head, with head AIS ≥ 3).

## Figures and Tables

**Table 1:** Comparison of demographic characteristics of included patients.

Characteristics	Total Patients	Survival	Mortality	*p*-Value
Patient number	15,164 (100%)	15,019 (99%)	145 (1%)	
Age (years)				<0.001
Age < 65 years old	8890 (58.6%)	8837 (58.8%)	53 (36.6%)	
Age ≥ 65 years old	6272 (41.4%)	6180 (41.2%)	92 (63.4%)	
Sex, n (%)				<0.001
Female	7520 (49.6%)	7479 (49.8%)	41 (28.3%)	
Male	7644 (50.4%)	7540 (50.2%)	104 (71.7%)	
Vital sign				
SBP	142 (124–161)	142 (124–161)	146 (109.5–174)	0.864
DBP	83 (73–94)	83 (73–94)	84 (66–99)	0.332
RR	18 (16–20)	18 (16–20)	18 (16–20)	0.094
HR	84 (73–96)	84 (73–96)	89 (74–101)	0.023
Triage				<0.001
1	667 (4.4%)	595 (4.0%)	72 (49.7%)	
2	5908 (39.0%)	5866 (39.1%)	42 (29.0%)	
3	8444 (55.8%)	8413 (56.1%)	31 (21.4%)	
4 and 5	125 (0.8%)	125 (0.8%)	0 (0.0%)	
Injury severity				
RTS	7.84 (7.84–7.84)	7.84 (7.84–7.84)	6.90 (5.00–7.84)	<0.001
RTS < 7	668 (4.4%)	594 (4.0%)	74 (51.4%)	<0.001
ISS	9 (4–9)	9 (4–9)	25 (9–29)	<0.001
ISS ≥ 16	1125 (7.4%)	1027 (6.8%)	98 (67.6%)	<0.001
Trauma team activation	495 (3.3%)	444 (3.0%)	51 (35.2%)	<0.001
Time for surgery	444 (248–848)	446 (251–850)	240 (116.5–473)	<0.001
Traumatic brain injury				<0.001
Non-TBI	14,151 (93.3%)	14,091 (93.8%)	60 (41.4%)	
TBI	1013 (6.7%)	928 (6.2%)	85 (58.6%)	
Injury type				0.080
Penetrating injury	807 (5.3%)	804 (5.4%)	3 (2.1%)	
Non-penetrating injury	14,357 (94.7%)	14,215 (94.6%)	142 (97.9%)	
Mechanism of injury				0.002
Traffic road injury	5112 (37.7%)	5045 (37.6%)	67 (51.5%)	
High fall	1370 (10.1%)	1353 (10.1%)	17 (13.1%)	
Low fall	5403 (39.9%)	5362 (39.9%)	41 (31.5%)	
Others	3279 (21.6%)	3259 (21.7%)	20 (13.8%)	
Comorbidity				
CNS diseases	672 (4.4%)	66 (4.4%)	6 (4.1%)	0.863
CVD	3965 (26.1%)	3925 (26.1%)	40 (27.6%)	0.692
CKD	351 (2.3%)	339 (2.3%)	12 (8.3%)	0.003
Diabetes mellitus	1676 (11.1%)	1653 (11.0%)	23 (15.9%)	0.250
Hospitalization				
Total LOS days	7 (4–10)	7 (4–10)	10 (4–22.5)	<0.001
Total LOS ≥ 30 days	687 (4.5%)	661 (4.4%)	26 (17.9%)	<0.001
ICU admission	1818 (12.0%)	1695 (11.3%)	123 (84.8%)	0.040
ICU LOS, days	5 (3–11)	5 (3–11)	6 (3–19)	<0.001
ICU LOS ≥ 14 days	369 (22.0%)	328 (21.1%)	41 (33.3%)	0.002
In-hospital mortality	145	---	145	----
Death within 24 h	10 (6.9%)	----	10 (6.9%)	----

Abbreviations: SBP, systolic blood pressure; DBP, diastolic blood pressure; RR, respiratory rate; HR, heart rate; CNS diseases, central nervous system diseases; CKD, chronic kidney disease; CVD, cardiovascular disease; ISS, injury severity score; RTS, revised trauma score; TBI, traumatic brain injury; LOS, length of stay; ICU, intensive care unit. The continuous variables are presented as median and interquartile range (IQR).

**Table 2:** Comparison of waiting emergent surgery times (WESTs) among all patients across various subgroups.

Subgroup	Median (IQR)	*p*-Value
WEST per 30 min		
WEST < 30 min	16 (9–23)	<0.001
30 ≤ WEST < 60 min	48 (38–55)	
60 ≤ WEST < 90 min	76 (70–84)	
WEST ≥ 90 min	468 (275–873)	
Age		
Age < 65 years old	419 (231–802)	<0.001
Age ≥ 65 years old	478 (276–905)	
RTS		
RTS < 7	301 (136–760)	<0.001
RTS ≥ 7	450 (255–849)	
ISS		
ISS < 16	447 (256–840)	<0.001
ISS ≥ 16	397 (155–948)	
Major injury site		
Head AIS ≥ 3	413 (157–956)	<0.001
Chest AIS ≥ 3	492 (197–1077)	
Abdominal AIS ≥ 3	353 (156–946)	
Hospitalization		
Total LOS < 30 days	446 (252–847)	<0.001
Total LOS ≥ 30 days	406 (182–881)	
ICU admission		
Yes	432 (189–982)	0.273
No	445 (256–830)	
In-hospital mortality		
Yes	240 (116–473)	<0.001
No	446 (251–850)	

Abbreviations: WEST, waiting emergent surgery time; SBP, systolic blood pressure; DBP, diastolic blood pressure; RR, respiratory rate; HR, heart rate; ISS, injury severity score; AIS, abbreviated injury scale; RTS, revised trauma score; TBI, traumatic brain injury; LOS, length of stay; ICU, intensive care unit.

**Table 3:** Comparison of waiting emergent surgery time (WEST) among trauma patients receiving emergent surgery within 2 h across various subgroups.

Subgroup	Median (IQR)	*p*-Value
Age		
Age < 65 years old	81 (56–99.75)	<0.001
Age ≥ 65 years old	72 (40–99)	
RTS		
RTS < 7	89 (70.5–102)	<0.001
RTS ≥ 7	76 (48–99)	
ISS		
ISS < 16	76 (47–99)	<0.001
ISS ≥ 16	88 (64–103)	
Major injury site		
Head AIS ≥ 3	91 (72–105)	<0.001
Chest AIS ≥ 3	77 (53–93)	
Abdominal AIS ≥ 3	81 (60.5–96)	
Hospitalization		
Total LOS < 30 days	78 (49–99)	0.174
Total LOS ≥ 30 days	87 (60–99)	
ICU admission		
Yes	87 (58.75–100)	<0.001
No	76 (48–99)	
In-hospital mortality		
Yes	85 (62–98)	0.463
No	78 (50–99)	

Abbreviations: WEST, waiting emergent surgery time; SBP, systolic blood pressure; DBP, diastolic blood pressure; RR, respiratory rate; HR, heart rate; ISS, injury severity score; AIS, abbreviated injury scale; RTS, revised trauma score; TBI, traumatic brain injury; LOS, length of stay; ICU, intensive care unit.

**Table 4:** Results of multivariable logistic regression analysis of in-hospital mortality, ICU admission, and prolonged hospital stay (≥30 days).

Variables	In-Hospital Mortality		ICU Admission		Prolonged Hospital Stay ≥ 30 Days	
	Adjusted OR (95% CI)	*p*-Value	Adjusted OR (95% CI)	*p*-Value	Adjusted OR (95% CI)	*p*-Value
WEST per 30 min						
WEST < 30 min	Reference	---	Reference	---	Reference	---
30 ≤ WEST< 60 min	1.05 (0.17–6.35)	0.955	0.85 (0.41–1.80)	0.678	2.93 (0.92–9.33)	0.068
60 ≤ WEST< 90 min	1.12 (0.22–5.70)	0.890	0.62 (0.30–1.28)	0.199	1.80 (0.58–5.53)	0.307
WEST ≥ 90 min	0.60 (0.13–2.74)	0.510	0.70 (0.40–1.24)	0.225	1.69 (0.59–4.83)	0.324
Age						
Age < 65 years old	Reference	---	Reference	---	Reference	---
Age ≥ 65 years old	3.62 (2.44–5.35)	<0.001	1.84 (1.59–2.13)	<0.001	1.22 (1.01–1.46)	0.038
Sex						
Male	Reference	Ref	Reference	---	Reference	Ref
Female	0.47 (0.31–0.70)	<0.001	0.67 (0.58–0.77)	<0.001	0.83 (0.70–0.99)	0.042
Major injury or not						
RTS score						
RTS < 7	5.15 (3.32–7.99)	<0.001	5.97 (4.58–7.78)	<0.001	2.75 (2.16–3.51)	<0.001
RTS ≥ 7	Reference	---	Reference	---	Reference	---
ISS score						
ISS < 16	Reference	---	Reference	---	Reference	---
ISS ≥ 16	8.29 (4.54–15.1)	<0.001	20.3 (16.5–25.0)	<0.001	5.74 (4.39–7.50)	<0.001
TBI status						
No TBI	Reference	---	Reference	---	Reference	---
TBI	1.59 (0.91–2.81)	0.106	9.63 (7.66–12.1)	<0.001	1.90 (1.43–2.52)	<0.001
Injury type						
Blunt injury	Reference	---	Reference	---	Reference	---
Penetrating injury	1.36 (0.38–4.90)	0.636	0.48 (0.34–0.67)	<0.001	1.28 (0.86–1.90)	0.223
Mechanism						
Road traffic injury	Reference	---	Reference	---	Reference	---
High fall	1.09 (0.61–1.93)	0.776	0.77 (0.60–0.98)	0.033	1.25 (0.95–1.63)	0.106
Low fall	0.98 (0.61–1.55)	0.920	0.55 (0.46–0.66)	<0.001	0.66 (0.52–0.84)	0.001
Others	0.70 (0.39–1.26)	0.234	1.78 (1.49–2.12)	<0.001	1.13 (0.89–1.44)	0.304

Abbreviations: OR: odds ratio; CI: confidence interval; RTS: revised trauma score; ISS: injury severity score; TBI: traumatic brain injury.

## Data Availability

The data that support the findings of this study are available from the corresponding author upon reasonable request.

## Supplementary Materials

The following supporting information can be downloaded at: https://www.mdpi.com/article/10.3390/medicina60060960/s1, Table S1: STROBE Statement of current cohort study; Table S2: Comparison of demographic characteristics of included patients and patients with missing values.

## Abbreviations

WEST	Waiting emergent surgery time
ICU	Intensive care unit
LOS	Length of stay
IQR	Interquartile range
aOR	Adjusted odds ratio
CI	Confidence interval
HR	Heart rate
SBP	Systolic blood pressure
DBP	Diastolic blood pressure
RR	Respiratory rate
ISS	Injury severity score
RTS	Revised trauma score
TBI	Traumatic brain injury
AIS	Abbreviated injury score
CNS diseases	Central nervous system diseases
CKD	Chronic kidney disease
CVD	Cardiovascular disease
