# Supplementary material for: Association between Time to Emergent Surgery and Outcomes in Trauma Patients: A 10-Year Multicenter Study
Source: Medicina (Kaunas). 2024 Jun 10;60(6):960. doi: 10.3390/medicina60060960 (PMC11205494; doi:10.3390/medicina60060960)
Supplement: Supplementary file 1 [file medicina-60-00960-s001.zip › medicina-3026405-supplementary.pdf]

medicina

MDPI

Supplementary Table S1. STROBE Statement of current cohort study

	Item No	Recommendation	Section/paragraph
**Title and abstract**	1	(*a*) Indicate the study's design with a commonly used term in the title or the abstract	Title page (Page 1)
		(*b*) Provide in the abstract an informative and balanced summary of what was carried out and what was found	Abstract
**Introduction**			
Background/rationale	2	Explain the scientific background and rationale for the investigation being reported	Introduction, paragraph 1-3
Objectives	3	State specific objectives, including any prespecified hypotheses	Introduction, paragraph 4
**Methods**			
Study design	4	Present key elements of study design early in the paper	Method- 2.1. Study design and setting
Setting	5	Describe the setting, locations, and relevant dates, including periods of recruitment, exposure, follow-up, and data collection	Method- 2.1. Study design and setting
Participants	6	(*a*) Give the eligibility criteria, and the sources and methods of selection of participants. Describe methods of follow-up	Method- 2.2. Participant selection
		(*b*) For matched studies, give matching criteria and number of exposed and unexposed	N/A
Variables	7	Clearly define all outcomes, exposures, predictors, potential confounders, and effect modifiers. Give diagnostic criteria, if applicable	Method- 2.3. Variable measurements
Data sources/ measurement	8*	For each variable of interest, give sources of data and details of methods of assessment (measurement). Describe comparability of assessment methods if there is more than one group	N/A
Bias	9	Describe any efforts to address potential sources of bias	N/A
Study size	10	Explain how the study size was arrived at	N/A (use of all available data)
Quantitative variables	11	Explain how quantitative variables were handled in the analyses. If applicable, describe which groupings were chosen and why	Method- 2.3. Variable measurements
Statistical methods	12	(*a*) Describe all statistical methods, including those used to control for confounding	Method- 2.5. Statistical analysis

		(*b*) Describe any methods used to examine subgroups and interactions	Method-2.5. Statistical analysis
		(*c*) Explain how missing data were addressed	Method-2.2. Participant selection
		(*d*) If applicable, explain how loss to follow-up was addressed	N/A
		(*e*) Describe any sensitivity analyses	Method-2.5. Statistical analysis
**Results**			
Participants	13*	(a) Report numbers of individuals at each stage of study—e.g., numbers potentially eligible, examined for eligibility, confirmed eligible, included in the study, completing follow-up, and analysed	Results-3.1. Characteristics of study participants
		(b) Give reasons for non-participation at each stage	Results-3.1. Characteristics of study participants
		(c) Consider use of a flow diagram	Figure 1
Descriptive data	14*	(a) Give characteristics of study participants (e.g., demographic, clinical, social) and information on exposures and potential confounders	Results-3.1. Characteristics of study participants
		(b) Indicate number of participants with missing data for each variable of interest	Figure 1 Supplement Figure 1.
		(c) Summarize follow-up time (e.g., average and total amount)	N/A
Outcome data	15*	Report numbers of outcome events or summary measures over time	Results-3.1. Characteristics of study participants Table 1
Main results	16	(*a*) Give unadjusted estimates and, if applicable, confounder-adjusted estimates and their precision (e.g., 95% confidence interval). Make clear which confounders were adjusted for and why they were included	Results-3.3. Association between WEST

			and clinical outcomes Table 4.5.
		(*b*) Report category boundaries when continuous variables were categorized	Table 1
		(*c*) If relevant, consider translating estimates of relative risk into absolute risk for a meaningful time period	N/A
Other analyses	17	Report other analyses carried out—e.g., analyses of subgroups and interactions, and sensitivity analyses	Results-3.3. Association between WEST and clinical outcomes Table 4.5.
**Discussion**			
Key results	18	Summarize key results with reference to study objectives	Discussion, paragraph 1
Limitations	19	Discuss limitations of the study, taking into account sources of potential bias or imprecision. Discuss both direction and magnitude of any potential bias	Discussion, paragraph 5
Interpretation	20	Give a cautious overall interpretation of results considering objectives, limitations, multiplicity of analyses, results from similar studies, and other relevant evidence	Discussion, paragraph 2-4
Generalizability	21	Discuss the generalizability (external validity) of the study results	Page 5
**Other information**			
Funding	22	Give the source of funding and the role of the funders for the present study and, if applicable, for the original study on which the present article is based	already reported in submission form

* Give information separately for exposed and unexposed groups.

Supplementary Table S2. Comparison of demographic characteristics of included patients and patients with missing values.

**Characteristics**	Missing number (%)	Included patients	Patient with missing value	P-value
Patient number	-----	15164(98.3%)	266(1.7%)	
Age (years)	2(0.0%)			0.083
Age <65 ys	-----	8890(58.6%)	170(63.9%)	
Age ≥65 ys	-----	6272(41.4%)	96(36.1%)	
Sex, n (%)	0 (0.0%)			0.070
Female	-----	7520(49.6%)	117(44.0%)	
Male	-----	7644(50.4%)	149(56.0%)	
Vital sign				
SBP	13(0.1%)	142(124-161)	140(119-159)	0.023
DBP	13(0.1%)	83(73-94)	80(68.5-91)	0.001
RR	32(0.3%)	18(16-22)	18(18-20)	0.070
HR	12(0.1%)	84(73-96)	85(73.5-97)	0.493
Triage	22(0.1%)			<0.001

1	-----	667(4.4%)	24(9.1%)	
2	-----	5908(39.0%)	171(64.8%)	
3	-----	8444(55.8%)	67(25.4%)	
4 and 5	-----	125(0.8%)	2(0.8%)	
Injury severity				
RTS	11(0.1%)	7.84(7.84-7.84)	7.84(7.84-7.84)	0.001
RTS < 7	11(0.1%)	668(4.4%)	20(7.5%)	0.015
ISS	8(0.1%)	9(4-9)	9(4-9)	0.007
ISS ≥ 16	8(0.1%)	1125(7.4%)	34(12.8%)	0.001
Traumatic brain injury	0(0.0%)			<0.001
Non-TBI	-----	14151(93.3%)	228(85.7%)	
TBI	-----	1013(9.7%)	38(14.3%)	
Injury type	0(0.0%)			0.996
Penetrative	-----	807(5.3%)	14(5.3%)	
Non-penetrative	-----	14357(94.7%)	252(94.7%)	
Mechanism of injury	0(0.0%)			0.126
Traffic road injury	-----	5112(37.7%)	99(40.7%)	
High fall	-----	1370(10.1%)	14(5.8%)	
Low fall	-----	5403(39.9%)	100(41.2%)	
Others	-----	3279(21.6%)	53(19.9%)	
Comorbidity	0(0.0%)			
CNS diseases	-----	672(4.4%)	22(8.3%)	0.003
CVD	-----	3965(26.1%)	61(22.9%)	0.237
CKD	-----	364(2.4%)	13(4.9%)	0.041
Diabetes mellitus	-----	1676(11.1%)	26(9.8%)	0.553
Hospitalization				
Total LOS days	5(0.1%)	7(4-10)	7(4-11)	0.434
Total LOS ≥ 30 days	5(0.1%)	687(4.50%)	28(10.5%)	<0.001
ICU admission	5(0.1%)	1818(12.0%)	59(22.2%)	<0.001
ICU LOS, days	0(0.0%)	5(3-11)	6(3-19)	0.107
ICU LOS ≥ 14 days	0(0.0%)	369(22.0%)	20(33.9%)	0.031
In-hospital mortality	55(0.4%)	145(1.0%)	1(0.5%)	0.473

Abbreviations: SBP, systolic blood pressure; DBP, diastolic blood pressure; RR, respiratory rate; HR, heart rate; CNS diseases, central nervous system diseases; CKD, chronic kidney disease; CVD, cardiovascular disease; ISS, injury severity score; RTS, revised trauma score; LOS, length of stay; ICU, intensive care unit
